# The Influence of Social Comparison on Visual Representation of One's Face

**DOI:** 10.1371/journal.pone.0036742

**Published:** 2012-05-25

**Authors:** Ethan Zell, Emily Balcetis

**Affiliations:** 1 Department of Psychology, University of North Carolina at Greensboro, Greensboro, North Carolina, United States of America; 2 Department of Psychology, New York University, New York, New York, United States of America; Royal Holloway, University of London, United Kingdom

## Abstract

Can the effects of social comparison extend beyond explicit evaluation to visual self-representation—a perceptual stimulus that is objectively verifiable, unambiguous, and frequently updated? We morphed images of participants' faces with attractive and unattractive references. With access to a mirror, participants selected the morphed image they perceived as depicting their face. Participants who engaged in upward comparison with relevant attractive targets selected a less attractive morph compared to participants exposed to control images (Study 1). After downward comparison with relevant unattractive targets compared to control images, participants selected a more attractive morph (Study 2). Biased representations were not the products of cognitive accessibility of beauty constructs; comparisons did not influence representations of strangers' faces (Study 3). We discuss implications for vision, social comparison, and body image.

## Introduction

People frequently compare themselves to others [1], [2]. For example, people compare their salary to their co-worker's salary [3] and their physical fitness to that of professional athletes [4]. Much research suggests that social comparison influences self-evaluations [5] as expressed through explicit and implicit self-judgments [6]–[7]. However, the current research goes beyond to ask if social comparison influences processes considered more primary than explicit or implicit self-evaluation. Just as social comparison influences cognitive self-judgments, this research asks if social comparison similarly influences the way people come to see themselves. This research seeks to explore whether social comparison changes how people come to form perceptual representations of their own faces.

To our knowledge, no research has examined the effect of social comparison on visual self-representation, an outcome that can be differentiated from self-judgment in a variety of ways. First, face recognition occurs much more quickly than explicit cognitive judgments [8]–[10]. Second, face perception can occur without conscious awareness and requires few if any attentional resources [11]–[15]. Much research in social judgment examines the effects of comparison on traits that are inherently ambiguous [16]. Social judgment is subjective because traits lack verifiability [17] and must be constructed through processes implicating memory [18]. In contrast, face recognition is less malleable because one's face is a concrete and verifiable feature that most people see often. Thus, reality constrains the malleability of visual representation [19]. Although social comparison is a potent and pervasive process, it remains to be seen whether its effects extend to visual representation of the self—an outcome that is concrete, objectively verifiable, and highly familiar. In sum, because visual self-representation differs from explicit self-evaluation in multiple ways, still open is the question of whether social comparison can exert an influence on the recognition of one's own face.

Understanding the consequences of social comparison on visual self-representation is critical given that lower-level processes are often the building blocks upon which higher-level cognition and action are based. For example, perceived attractiveness of oneself predicts the types of romantic partners people commit to [20]. In addition, self-perceptions of attractiveness can lead to maladaptive behaviors including the modification of one's appearance through restrictive eating behaviors [21]–[22] and invasive cosmetic procedures [23]. Because higher order cognitive judgment and behavioral choices can be the product of lower-level perceptual processes, we examined the degree to which visual representation of one's own face is subject to influence by social comparison processes.

This investigation uses as a basis for prediction research, which suggests that people's cognitive evaluations of their own bodies are malleable and subject to influence by social comparison processes. For instance, exposure to photographs of attractive, same-gender targets led people to explicitly evaluate themselves as less attractive [24]–[25]. Similarly, female adolescents expressed greater dissatisfaction with their bodies if they exhibited more intense “celebrity worship” suggesting comparison to extreme standards depicted in the media affects evaluations of one's body [26]. Conversely, exposure to photographs of people who failed to meet ideal standards of beauty led people to express less dissatisfaction with their own bodies [27]. Explicit judgments about oneself and one's appearance are strongly affected by social comparison processes.

Beyond cognitive self-evaluation, preliminary evidence suggests that visual representation of oneself appears to be flexible even though people gain objective information about their appearance every time they catch their own reflections [28]. Existing evidence suggests that visual self-representation depends both on the internal qualities of the perceiver and external constraints of the situation. For example, implicit self-esteem predicted the attractiveness of the image participants selected as depicting their actual likeness [29]. In addition, people were more likely to select a photograph as the one depicting their actual likeness when that photograph had been morphed with a trustworthy rather than untrustworthy target [30]. Beyond internal states of the perceiver, visual representations of the self depend on the external context. Participants previously exposed to images of moderately overweight models selected a more rotund line drawing as reflecting their appearance than participants exposed to standard models or control stimuli [31]. Thus, converging lines of evidence suggest that visual self-representation is malleable.

However, it is unclear what component of self-representation is subject to influence because previous tests did not utilize a mirror when selecting the image that depicts their likeness. Asking participants to select an image they believe depicts themselves without requiring participants to look at themselves or use a mirror requires that measures of self-representation rely solely on memory. People must recall the shape of their body or the features of their face, for instance, in order to select the image that reflects their likeness. While people may be motivated and in fact believe that they are objectively selecting the correct image, they may in fact be drawn to a positively skewed image after engaging in a biased search through memory for other photographs that similarly depict a more positive rendition of themselves [19], [32]–[33]. Classic research that tests the effects of social psychological processes on visual representation, too, has traditionally experienced difficulty in ruling out the role of memory when testing visual phenomena [34]–[36].

Therefore, it still remains unclear whether situational factors influence processes considered more basic than judgment, such as visual representation of the self when looking into a mirror. The current research sought to explore this possibility, and specifically to test a novel question: can social comparison influence representations of one's own face? Even when looking at themselves in a mirror, comparison standards should impact how people view their own face. Specifically, we predicted that comparing oneself to more attractive standards would lead people to see their own face as less attractive whereas comparing to less attractive standards would lead people to see their own face as more attractive. That is, we predicted that social comparison would produce contrast effects in visual representation of one's own face.

We expected that visual self-representation would be contrasted against the comparisons available for two reasons. First, in these studies, we presented participants with relevant comparisons that were distinct (Studies 1–3) and extreme (Study 1). When comparisons reflect extreme [37] and distinct information about a specific person [38], self-evaluation is likely to be contrasted from the standard. For instance, reading, “John is a millionaire” is likely to produce a self-evaluation of, “I am poor.” When evaluating one's appearance, exposing participants to an image of a slim rather than overweight female leads them to feel less attractive and less satisfied with their appearance [39]. Conversely, when comparisons reflect abstract trait information, self-evaluation is likely to be assimilated toward the standard. For instance, the word “wealthy” is likely to produce a self-evaluation of, “I am wealthy.” When evaluating one's appearance, activating the trait “overweight” led people to recall their own bodies as being more overweight [31]. For this reason, we expected comparisons with distinct upward (Study 1) and downward (Study 2) comparison standards to produce contrast effects in visual self-representation.

In three studies, we explored the effects of social comparison on explicit self-judgment and visual representation of one's own face. All studies were approved by the Internal Review Boards at Ohio University, where the research was conducted. Study 1 tested whether upward social comparison involving exposure to photographs of highly attractive models that were relevant (i.e. same gender) led to more negative explicit self-evaluations of attractiveness and less attractive visual representation of one's face than exposure to irrelevant (i.e. opposite gender) comparison others. Study 2 tested whether explicit downward social comparison with unattractive peers influenced self-evaluations and visual self-representation when viewing oneself with the aid of a mirror. Finally, to refute an alternative explanation that cognitive accessibility of appearance-related concepts influenced self-evaluations and representation, Study 3 examined whether social comparisons influenced visual representation of the face of a stranger. We predicted that exposure to relevant social comparison targets would influence self-evaluations of one's own attractiveness and visual representations of one's own face but not evaluations or representations of another person. We expected these effects would occur even when looking at oneself in a mirror, thus receiving direct visual information, and would occur independent of implicit and explicit self-esteem.

## Study 1

Study 1 tested whether exposure to upward social comparison targets influences explicit self-evaluation and people's representations of their own face. We exposed participants to photographs of attractive professional models who were either relevant social comparison targets (i.e. same gender) or irrelevant social comparison targets (i.e. opposite gender) [24]. We predicted that participants exposed to attractive models would explicitly evaluate their own attractiveness less favorably and select an image of their own face that was less attractive than participants exposed to attractive, opposite gender models or control images.

### Methods

Upon arrival to the lab, we photographed 36 participants' (22 female) faces. Participants completed the Rosenberg Self-Esteem scale to measure explicit self-esteem [40] and rated how much they liked their own name to measure implicit self-esteem [41]; both measures were included as covariates in later analyses.

Then, through a test ostensibly measuring “aesthetic judgment,” participants randomly received one of three social comparison manipulations. Participants assigned to the *same-gender comparison condition* viewed 20 headshots of attractive professional models of the same gender. This condition served as an upward social comparison condition, because same-gender targets are considered relevant comparisons. Participants assigned to the *opposite-gender control condition* viewed 20 headshots of models of the opposite gender. This condition served as a control condition, as opposite-gender targets are irrelevant comparisons [24]. Also, this condition served as a control to test whether exposure to faces in and of themselves influence visual self-representations. As a manipulation check, participants evaluated the target's attractiveness on 1 (*not at all*) to 7 (*extremely*) scales. Pre-testing with a separate group of participants (*n* = 20) showed that the same-gender and opposite-gender photographs did not differ in attractiveness, *p*>.20. Finally, participants in the *no comparison control condition* viewed pictures of mundane landscapes and rated how beautiful the landscapes were on the same scales. This condition served as a baseline evaluation in the absence of information about people in general. After viewing and rating all 20 images, participants provided explicit self-evaluations of their own attractiveness. Using a 1 (*not at all*) to 7 (*extremely*) scale, participants indicated how attractive they felt, satisfied they felt about their appearance, and how satisfied they felt in general (α = .89).

**Figure 1:**
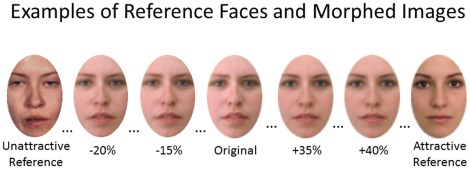
Reference faces with which participants' original photographs were morphed and selected examples of morphed images. Morphed images were presented in a random order during the visual self-representation task; reference faces were not presented to participants.

Then, participants completed the visual self-representation measure, labeled for participants as the “perceptual accuracy test.” We created morphed images by using an oval crop of the participant's original face photograph that excluded their hair and ears [28]–[30]. Then, we morphed the participant's face with 2 reference faces using Abrosoft's Fantamorph computer program. The attractive reference was a beautiful, artificially created composite face that was highly symmetrical. The unattractive reference was the face of a person suffering from a facial disorder (i.e., craniofacial syndrome) that was highly asymmetrical. We chose these references to replicate previously used methodologies [29]. We created 12 morphed images that reflected 5% increment increases of either the attractive or unattractive reference face. For example, the +40% image was created by morphing participants' original photograph with 40% of the attractive one, while the −20% image was created by morphing participants' original with 20% of the unattractive photograph (see Figure 1).

Participants were presented with a total array that included 13 images presented simultaneously on a 15-inch computer monitor. In a scattered, random order appearing across all areas of the computer screen, participants saw their actual photograph, 8 morphs with the attractive reference (+5% to +40%), and 4 with the unattractive reference (−5% to −20%). We used this range because previous research demonstrated that participants rarely selected faces outside of it as their own [29]. To measure visual self-representation, participants told the experimenter which photograph they thought matched the image they saw when looking into the mirror [42]. Participants were directed to use a mirror to ensure that face selection reflected perceptual representation processes, as opposed to memory based processes exclusively. All participants indicated in debriefing that they utilized the mirror to make a selection. Furthermore, during pilot testing, participants were covertly observed during the visual self-representation task; all participants utilized the mirror to select a morphed image, suggesting the instructions were likely to induce use of the mirror in the main study.

Following these procedures, participants underwent debriefing and probes for suspicion of the hypotheses. In all studies, no participant identified the connection between the social comparison and visual self-representation tasks.

### Results

Evaluations of same-gender (α = .85, M = 5.76, SD = 0.87) and opposite-gender photographs (α = .85, *M* = 5.53, *SD* = 0.78) were substantially above the midpoint of the scale (*t*s<.001), demonstrating that they were viewed as highly attractive targets.

Social comparison condition influenced explicit self-evaluations regarding one's own attractiveness, *F*(2, 33) = 3.58, *p*<.05, *η* ^2^ = .18. Participants in the *same-gender comparison condition* reported lower self-evaluations than participants in the *opposite-gender*, *t*(33) = 2.06, *p*<.05, *d* = 0.77, and *no comparison control* conditions, *t*(33) = 2.51, *p*<.05, *d* = 1.01, see Table 1.

**Table 1:** Means (SDs) for Explicit Self-Ratings and Percent Morphed Face Selected During Visual Self-Representation for Study 1.

	Same-Gender	Opposite-Gender	Landscape Control
Explicit Self-Ratings	4.2_a_ (1.1)	4.9_b_ (0.9)	5.1_b_ (0.7)
Visual Self-Representation	−0.4_a_ (9.4)	13.8_b_ (16.0)	12.5_b_ (13.6)

*Note*: Subscripts that differ within rows indicate significance at *p*<.05.

Social comparison also influenced visual representation of one's own face, *F*(2, 33) = 4.20, *p*<.05, *η* ^2^ = .20. Participants in the *same-gender comparison condition* selected a morphed face that was less attractive than participants in the *opposite-gender*, *t*(33) = 2.62, *p*<.05, *d* = 1.08, and *no comparison control* conditions, *t*(33) = 2.39, *p*<.05, *d* = 1.11, see Table 1.

Although our primary hypotheses concerned the effects of social comparison on self-evaluations and visual self-representations, we tested for the effects of other predictor variables. We reran the models predicting self-evaluation and visual self-representation from social comparison condition (coded same gender = −2, no comparison control = +1, opposite gender = +1) and added participant gender (coded female = 1, male = 0), implicit self-esteem, and explicit self-esteem as predictors. The effect of social comparison condition on explicit self-evaluation remained significant when all other predictors were simultaneously entered into the model, β = .43, *t* = 3.80, *p* = .001. Explicit self-esteem was a significant predictor of explicit self-evaluation, β = .54, *t* = 4.59, *p*<.001, but implicit self-esteem, β = .23, *t* = 1.93, *p* = .06, and gender, β = .21, *t* = 1.81, *p* = .08, did not significantly predict explicit self-evaluation.

Further, the effect of social comparison condition on visual self-representation also remained significant when all other predictors were entered, β = .46, *t* = 2.91, *p* = .007. Explicit self-esteem, β = .02, *t* = 0.12, *p* = .91, implicit self-esteem, β = .02, *t* = 0.10, *p* = .92, and gender, β = .16, *t* = 1.01, *p* = .32, did not significantly predict visual self-representation.

Finally, we tested whether visual self-representations were produced as a result of changes in explicit self-ratings or whether they were statistically independent. While there was a marginally significant correlation between the two outcome variables (*r* = .32, *p* = .06), a sobel test [43] showed that the effect of social comparison (dummy coded as −2 for same-gender comparison, and +1 for opposite-gender and no comparison control) on visual self-representation was not mediated by explicit self-ratings, *z* = .84, *p* = .40. This analysis suggests that visual representations were not the result of changes in explicit self-evaluation judgments.

## Study 2

While Study 1 exposed participants to upward comparison targets that were either relevant or irrelevant, Study 2 tested whether relevant downward social comparisons influence visual self-representation. Additionally, although incidental exposure to comparison targets does induce comparison processes [24]–[25], we still were left to infer that the act of comparison occurred. In Study 2, we explicitly directed participants to compare their appearance to the appearance of the provided targets to ensure that social comparison processes were activated and produced the same effects on evaluation and representation. Finally, although unlikely, it is possible that in Study 1 participants differentially responded to the social comparison conditions as a function of their own level of attractiveness. To explore this possibility, outside observers rated the attractiveness of each participant. We included objective evaluations of participant's attractiveness in the analyses to ensure that the obtained social comparison effects are not due to pre-existing differences in attractiveness.

### Methods

We photographed 24 participants (17 female) and asked them to complete the same implicit and explicit self-esteem scales as in Study 1. Then, participants randomly received one of two social comparison manipulations. Participants assigned to the *lateral comparison condition* viewed 20 photographs of same-gender peers who were moderately attractive, while participants assigned to the *downward comparison condition* viewed 20 photographs of unattractive same-gender peers. These photographs were cropped to focus primarily on the face. The photos were taken from HotorNot.com, a website on which each photograph had been rated by several hundred people on a 1 (*not hot*) to 10 (*hot*) scale. Pre-testing with a separate group of participants (*n* = 46) indicated that the downward comparison targets were rated as less attractive than the lateral comparison targets, *p*<.001.

After viewing each photograph, participants compared the target's attractiveness to their own by responding to the following question: “How attractive is this person in comparison to you?” Comparative evaluations were made on 1 (*much less attractive than me*) to 7 (*much more attractive than me*) scales. After the comparison task, all participants provided self-evaluations of their own attractiveness on the same measures as Study 1 (α = .77) and completed a visual self-representation measure while seated in front of a mirror as was done in Study 1. During debriefing, all participants reported using the mirror to select a morphed image.

A second group of participants (*n* = 33) rated the attractiveness of each participant as depicted in their original photograph using a 0 (*not at all attractive*) to 10 (*very attractive*) scale. We averaged these ratings to obtain an objective evaluation of participant's actual attractiveness.

### Results

Explicit comparative evaluations about the photographed comparison other were less favorable relative to oneself in the *downward comparison condition* (*M* = 2.67, *SD* = 0.78) than in the *lateral comparison condition* (*M* = 3.96, *SD* = 0.39), *t*(22) = 5.14, *p*<.001, *d* = 2.19. Evaluations of downward comparison targets were significantly below the midpoint of the scale, which was 4, *t*(11) = 5.93, *p*<.05, yet evaluations of lateral comparison targets did not differ from the scale midpoint, *t*(11) = 0.33, *p* = .75. This confirms that the photographs used in the downward comparison condition depicted targets considered substantially less attractive than participants themselves, and the photographs used in the lateral condition equally attractive as participants themselves.

Social comparison condition influenced explicit self-evaluations of one's own attractiveness. Participants in the *downward comparison condition* reported higher explicit self-evaluations than participants in the *lateral comparison condition*, *t*(22) = 3.21, *p*<.01, *d* = 1.37, see Table 2.

**Table 2:** Means (SDs) for Explicit Self-Ratings and Percent Morphed Face Selected During Visual Self-Representation for Study 2.

	Lateral	Downward
Explicit Self-Ratings	4.9_a_ (0.8)	5.8_b_ (0.5)
Visual Self-Representation	−1.3_a_ (13.3)	11.7_b_ (9.4)

*Note*: Subscripts that differ within rows indicate significance at *p*<.05.

Social comparison also influenced visual representation of one's own face. Participants in the *downward comparison condition* selected a morphed face that was more attractive than participants in the *lateral comparison condition*, *t*(22) = 2.75, *p*<.05, *d* = 1.17, see Table 2.

As in Study 1, we tested for the effects of other predictor variables. We reran the models predicting self-evaluation and visual self-representation from social comparison condition (coded downward comparison = 1, lateral comparison = 0) and added participant gender (coded female = 1, male = 0), implicit self-esteem, explicit self-esteem, and participant's actual attractiveness as predictors. The effect of social comparison condition on explicit self-evaluation remained significant when all other predictors were simultaneously entered into the model, β = .48, *t* = 2.84, *p* = .01. Explicit self-esteem was a significant predictor, β = .42, *t* = 2.29, *p* = .04, but implicit self-esteem, β = .06, *t* = 0.35, *p* = .73, gender, β = .02, *t* = 0.09, *p* = .93, and participant's actual attractiveness, β = .17, *t* = 0.85, *p* = .41, did not significantly predict explicit self-evaluation.

Further, the effect of social comparison condition on visual self-representation also remained significant when all other predictors were entered, β = .41, *t* = 2.06, *p* = .05. Explicit self-esteem, β = .36, *t* = 1.66, *p* = .11, implicit self-esteem, β = .21, *t* = 1.01, *p* = .33, gender, β = .11, *t* = 0.52, *p* = .61, and participant's actual attractiveness, β = .02, *t* = 0.10, *p* = .92, did not significantly predict visual self-representation.

As in Study 1, explicit self-ratings and visual self-representations were statistically independent. That is, although the two outcome variables were significantly correlated (*r* = .50, *p*<.05), a sobel test [48] showed that the effect of social comparison on visual self-representation was not mediated by explicit self-ratings, *z* = 1.29, *p*>.15.

## Study 3

Study 3 addressed an alternative account for the findings obtained in Studies 1 and 2. One could argue that exposure to photographs of attractive and unattractive people primed or made accessible beauty-related concepts. Study 1 provided initial evidence that this was not the case. Beauty-related concepts should have been equally active in both the relevant, same-gender condition and in the irrelevant, opposite-gender condition as the photographs were rated to be equally attractive in both conditions. However, it is still possible that gender similarity differentially activated beauty-related concepts, or that the cognitive structure of facial beauty is organized according to gender.

To provide an additional test of this cognitive accessibility alternative explanation, Study 3 examined whether social comparison affected the representation of others. If exposure to unattractive people primes appearance-related concepts (e.g., ugly, unappealing), then exposure to unattractive peers should carry over to influence the representation of others, just as it influences representation of oneself [44]. Alternatively, the social comparison explanation we postulate predicts that exposure to unattractive peers should not influence the representation of others. While comparisons between the self and others changes self-evaluations, they should not influence evaluations of other people. This prediction is consistent with past work showing that social comparison [6] and concept-priming [31] more readily influence self-evaluation than evaluation of others.

Furthermore, rarely during social comparison do processes carry over to third parties not referenced in the comparison [45]. Specifically, comparing one's spouse to a highly attractive model may affect representations of the spouse, but should not affect self-representations. Similarly, comparing oneself to a highly attractive model may affect representations of oneself, but not one's spouse. Therefore, we predicted that exposure to social comparison targets would influence explicit self-evaluation but would not influence visual representation of others' faces.

### Methods

We yoked 24 new observer participants to each participant we photographed in Study 2. New observer participants were of the same race and gender as Study 2 participants to which they were matched, and came from the same participant pool. New observer participants experienced a procedure parallel to that of Study 2. Observers initially were photographed, completed measures of implicit and explicit self-esteem, and were randomly assigned to one of the two comparison conditions used in Study 2 in which they explicitly rated photos (lateral comparison, downward comparison). Immediately after the photo-rating task, observers provided explicit self-evaluations of their own appearance using the same measures as participants in Studies 1 and 2 (α = .84). It was necessary to have participants evaluate themselves *before* they evaluated and were exposed to the yoked target. Otherwise, the effect of social comparison on self-evaluation may have been contaminated by possible comparisons with the yoked target.

Then, observers were brought to an adjacent laboratory room and seated at a computer with a 20-inch monitor. First, observers saw an original, unaltered photograph of the participant from Study 2 to which they were yoked. The photograph was displayed on the right side of the computer screen and was the same size as the mirror image Study 2 participants saw of themselves. Thus, the image to which observers were exposed assumed the same basic shape, size, and location as the mirror image of themselves participants in Study 2 saw. Observers explicitly rated this person's appearance on the same measures Study 2 participants used to rate themselves (α = .76).

Finally, the experimenter presented observers with the morphed photo array of the participant from Study 2 to which they were yoked. This array appeared on the left side of the screen, and the unaltered original photo remained on the right side of the screen. The size of the morphed array assumed the same size as that presented to participants in Study 2. Observers reported which morphed image matched the unaltered photograph they viewed on the right side of the screen—the very same place where participants in Study 2 looked to see their own image in a mirror. During debriefing, all participants indicated that they used the unaltered photograph on the right side of the screen to select a morphed image.

### Results

In the manipulation check, explicit comparative evaluations were less favorable about the photographed comparison other relative to oneself in the *downward comparison condition* (*M* = 2.87, *SD* = 0.78) than in the *lateral comparison condition* (*M* = 3.93, *SD* = 0.87), *t*(22) = 3.16, *p* = .005, *d* = 1.35. Evaluations of downward comparison targets were significantly below the midpoint of the scale, *t*(11) = 5.05, *p*<.001, yet evaluations of the lateral comparison targets did not differ from the scale midpoint, *t*(11) = 0.26, *p* = .80. This serves as a manipulation check that the photographs served as lateral or downward social comparison targets.

Exposure to social comparison information had a significant influence on explicit self-evaluations. Participants in the *downward comparison condition* reported more positive self-evaluations of their own attractiveness than participants in the *lateral comparison condition*, *t*(22) = 2.29, *p*<.05, *d* = 0.98, see Table 3.

**Table 3:** Means (SDs) for Explicit Self and Other Ratings of Attractiveness and Percent Morphed Face Selected During Visual Target Representation for Study 3.

	Lateral	Downward
Explicit Self-Ratings	4.9_a_ (0.8)	5.7_b_ (0.7)
Explicit Other-Ratings	5.0_a_ (1.1)	5.2_a_ (0.4)
Visual Other-Representation	4.6_a_ (13.9)	5.4_a_ (17.9)

*Note*: Subscripts that differ within rows indicate significance at *p*<.05.

We also tested for the effects of other predictor variables. We reran the model predicting self-evaluation from social comparison condition (coded downward comparison = 1, lateral comparison = 0) and added participant gender (coded female = 1, male = 0), implicit self-esteem, and explicit self-esteem as predictors. The effect of social comparison condition on explicit self-evaluation largely remained when all other predictors were simultaneously entered into the model, β = .32, *t* = 1.84, *p* = .08. Explicit self-esteem was a significant covariate, β = .52, *t* = 3.12, *p* = .006, but implicit self-esteem, β = .14, *t* = 0.84, *p* = .41, and gender, β = .20, *t* = 1.19, *p* = .25, did not significantly predict explicit self-evaluation.

Exposure to social comparison information did not affect explicit evaluations of the attractiveness of the yoked target, *t*(22) = 0.50, *p* = .60, *d* = 0.21, see Table 3. Social comparison condition also did not influence visual representations of the yoked target's face, *t*(22) = 0.13, *p* = .90, *d* = 0.05, see Table 3. In sum, social comparisons did not influence explicit evaluations or visual representation of others, suggesting the effects of social comparison did not carry over to evaluations of others.

Further, these findings rule out the possibility that the social comparison task was simply priming beauty-related concepts. If attractive faces primed cognitive constructs of beauty, even those that are organized within gender categories, activated constructs related to beauty should impact all subsequent judgments including self and other evaluations and visual representations of others. These data suggest that the photographs presented during the photo-rating task were extreme enough to impact self-evaluations, however they did not impact evaluations or visual representations of others. These data argue against the possibility that cognitive accessibility of beauty constructs was responsible for visual representations in these studies.

## Discussion

Do social comparison processes affect self-representations? The current research suggests, first, that social comparisons influence both self-evaluations and more basic processes including visual representation of one's face. Relevant upward social comparison, relative to irrelevant or no comparison, led people to evaluate themselves less positively and to visual represent themselves when looking into the mirror as less attractive (Study 1). Additionally, explicit downward comparison, relative to lateral comparison, led people to evaluate themselves more positively, and represent themselves as more attractive (Study 2). Thus, the effect of social comparison occurred both in situations when comparison processes were likely to occur but were not explicitly required of participants (Study 1) and when comparison was explicitly directed (Study 2). Social comparison processes influenced visual representation of one's own face even when participants had direct access to a mirror in which they all viewed their own face (Studies 1–2). In addition, social comparison processes influenced visual self-representation largely independent of implicit and explicit self-esteem (Studies 1–2) and participants' actual level of attractiveness (Study 2). This research suggests that visual representation of the self may be malleable and contextually dependent upon ongoing psychological processes.

It is unlikely that cognitive accessibility of beauty-related constructs influenced visual self-representation. First, exposure to attractive models did not unconditionally influence representation. Instead representation was influenced only when participants were exposed to attractive models that were relevant, same-gender social comparison targets (Study 1). Second, social comparison influenced self-evaluations (Studies 1–3) and visual representation of one's own face (Studies 1–2), but not visual representations of others' faces (Study 3). These data serve as the first demonstration that social comparison affects earlier perceptual processes including visual self-representation. Given that one's likeness is objectively verifiable, unambiguous, and people are updated on their own appearance every time they see their reflection, that comparison affects visual self-representation attests to the strength and pervasiveness of comparison processes.

### Self-Awareness and Visual Self-Representation

Critical to our design, participants provided measures of visual self-representation while seated in front of a mirror. Use of a mirror fundamentally transforms the self-representation task from one that relies solely on memory, to one that captures a combination of memory and perceptual-based processes. While the mirror creates visual, perceptual input, it can also increase self-awareness. Self-awareness is a psychological state in which people automatically compare themselves to internal standards and ideals, which can be unpleasant given that people often fail to match their ideals [46]. For example, looking into a mirror can draw attention to the blemishes on one's face. While the presence of mirrors can increase self-awareness, self-awareness is likely not confounded with social comparison processes in our studies. Since all participants regardless of comparison condition viewed themselves in front of a mirror, levels of self-awareness were equal across conditions. While self-awareness might be heightened in our studies compared to situations when mirrors are not present, self-awareness cannot be responsible for producing the differences in evaluation and representation we found among comparison conditions. However, future research could benefit by exploring the interactive effects of social comparison processes and self-awareness on visual self-representation.

### Accuracy in Self-Representation

It is possible that the static or moving nature of the target face impacted accuracy during the visual perception task. Participants in Study 3 viewed a static image of the target, while participants in Studies 1 and 2 viewed their image in a mirror. It is possible that static images made the face-matching task easier given the simpler perceptual input. However, this seems not to be the case. After exposure to lateral comparisons, participants in Study 2 selected an image of themselves that deviated −1.3% from their actual image while participants in Study 3 selected an image of others that deviated 4.6%. Given that participants were not more accurate when viewing static images of others compared to when viewing more complex moving images of themselves, it does not seem to be the case that decreased ease of perceptual processing contributes to the effects of social comparison on self-representation.

Further, across conditions, it may appear that participants' representations were generally inaccurate when choosing an image that depicted their actual likeness. Participants selected a morphed face that was somewhat more attractive than their actual image (Study 1: *M* = 8.61, one-sample *t* = 3.58, *p* = .001; Study 2: *M* = 5.21, one-sample *t* = 1.95, *p* = .06). One could interpret this data as indicating that people represent themselves in an overly favorable manner. During the face-selection process, however, we presented participants with more images of their face morphed with the attractive reference than images morphed with the unattractive reference. The range we chose reflected the range that participants actually used in previous research that employed the same dependent measure [29]. As a result, participants' odds of selecting a morphed image that was more attractive, as opposed to less attractive than their actual image, was greater just by mere chance. For this reason, the reported findings cannot be interpreted with respect to what constitutes an accurate selection, but instead differences can only be discussed with reference to relationships among manipulated conditions.

### Distinctiveness and Face Adaptation

Some readers may wonder about the effects of some aspects of our experimental paradigm, including distinctiveness of the comparison faces and exposure to other faces in general, on visual self-representation. First, the reference faces used to create the line up of morphed images of participants' own faces called upon attractive and unattractive references that were selected because they differed in facial symmetry, which is a key component of attractiveness [47]. It is possible that the reference faces differed on other factors including distinctiveness. For example, one could argue that the appearance of the target suffering from cranio-facial syndrome may be more distinct than that of the aggregated, highly attractive target. However, this possibility seems unlikely in light of past work showing that highly symmetrical faces are distinct and rare in the natural world [48]–[49]; most people to which others are exposed lack symmetry. Because symmetrical faces, like those used as our referent, are uncommon and unique outside artificial lab paradigms, symmetrical faces are distinct.

While we espoused that exposure to attractive and unattractive faces produces high level (i.e., cognitive) social comparison processes, other research suggests that exposure to attractive and unattractive faces leads to lower level face adaptation effects. According to face adaptation theories [50], exposure to relatively extreme faces shifts the perceived “average” level of attractiveness, which is used as a standard during representation of subsequent targets [51]–[52]. Thus, exposure to extreme faces produces perceptual contrast effects. Rather than competing processes, we believe that face adaptation effects are related to social comparison effects. Initial exposure to attractive and unattractive targets changes the standards that are available at low levels of processing when subsequently perceiving targets and making higher level assessments of representation. Thus face adaptation effects may contribute to social comparison phenomenon by shifting the standards that are available and which are used; psychometric functions might be employed to test this possibility in the future as we were not explicitly testing this perceptual mechanism.

Although exposure may shift low level perceptual standards as suggested by face adaptation theories, it is possible to dissociate the effects of social comparison theories and face adaptation theories. First, face adaptation theories suggest muted although still present contrast effects when there is a mismatch between the gender of the faces presented during preliminary exposure and the gender of the target faces [53]. That is, face adaptation theories might predict an effect of exposure to opposite-gender photographs on self-representation. On the other hand, social comparison theories might predict that opposite-gender faces presented during preliminary exposure should be considered irrelevant targets. Thus, social comparison theories predict no effect of exposure to opposite-gender faces on visual self-representation. As suggested by social comparison theories, self-representation was unaffected by exposure to opposite-gender photographs in Study 1.

Second, face adaptation theories suggest that contrast effects should be largest when targets are unfamiliar others [54]–[56] compared to when the target is the self [57]; [ but see 58]. However, social comparison theories suggest contrast effects should be larger when the target is the self compared to others. As supported by social comparison theories, exposure to unattractive others influence self-representation (Study 2) but did not influence representations of others (Study 3). Thus, it is possible that exposure to extreme faces shifts available low level standards which may be used, to varying degrees, during higher level social comparison processes particularly when those shifted standards are considered relevant. Further, these shifted standards may be considered relevant when forming representations of the self. While we are simply speculating on the relative influence of these two effects, future research could systematically disentangle the power of each to influence visual self-representation.

### Limitations and Future Directions

The present studies had a few limitations, which point the way for future research. First, Studies 1 and 2 had more female than male participants (65% female across studies) and relatively small sample sizes (60 participants across studies). Future research might balance participant gender and increase sample sizes to better test gender differences in response to social comparison during visual self-representation. While past research indicates that self-evaluative reactions to comparison others are generally comparable among men and women [5], it is possible that visual self-representation effects may be moderated by gender, given gender differences in issues related to body image. For instance, only an estimated 5 to 15 percent of people with anorexia or bulimia are male [59]. Gender differences may emerge if the ability to detect what might be smaller effects in men increased.

Another limitation in our paradigm was the fact that we presented participants with an unbalanced array of faces during visual self-representation measure. That is, we presented participants with more images of their face morphed with the attractive reference than images of their face morphed with the unattractive reference. Such unequal distributions offer participants greater visual experience with attractive images compared to unattractive images. We held this feature of the visual self-representation measure constant across social comparison conditions; therefore, unequal exposure cannot fully explain the effects of comparison conditions. Nonetheless, future research is needed to further explore whether our pattern of results would replicate using a balanced outcome where participants are presented with an equal number of attractive and unattractive references.

Finally, although it is unlikely that the present studies can be fully accounted for by a face adaptation explanation, additional research is needed to further explore whether exposure to attractive and unattractive targets influences self-representation as a function of social comparison processes, face-adaptation, or both. The results of Study 3, whereby exposure to unattractive targets did not affect representation of others, suggest that our results were likely driven by comparison and not adaptation. However, methodical limitations of Study 3, such as the use of a static image rather than a mirror image during target-perception, suggest that more work is needed to definitively rule out face adaptation as a contributing mechanism.

### Conclusion

It is important to investigate the scope of social comparison influences. By noting that comparison can exert an influence on early forms of information processing, including representation of one's own face, we take one step toward explaining why social comparison can be so deleterious, impacting even general levels of mental and physical health [60]–[61]. People may not be aware that social comparison processes are shaping the contents of their self-evaluations, and thus may lack the awareness or ability to control the effects of comparison [4]. If comparison goes unabated, as it might when comparison exerts an influence early on in information processing, then it is ever more possible that comparison can impact not just how people interpret and think about their social world but how they literally see it.
